# A Muddled Critic

**Published:** 1886-02

**Authors:** 


### A MUDDLED CRITIC.

Whence comes it that most of the world's great reformers choose for their hobbies matters of which they are most ignorant? The apostles of phoneticism are proverbially bad spellers, and temperance cranks usually the most intemperate of men—in their language. One of our respected exchanges, which, like Hamlet's uncle, is a thing of shreds and patches, and whose contents consist of far too large a percentage of pickings and pilferings, has an editor who seems determined to make innate philological genius serve in place of literary training and scholastic advantages. He is a good soul—a little straight-laced or so—but withal exceedingly captious and critical, and he presents to us less richly endowed mortals model sentences and precise examples in abundance. Here is a specimen of his ability as a linguist, and a good sample of his clear, flowing diction:

"Note your 'insult to the intelligence of the reader' in your 'liberal use of italics,' in speaking of Dr. Miller's views as expressed in the last International Medical Congress as given below."

Oh, shrine of Lindley Murray, once sacred, now profaned! What does all this mean? Reading this, can mortal man forbear the breaking out into exclamations!! *Italics,* or even SMALL CAPS? Is there any intelligence here to insult? *Is* the Medical Congress given below? DOES the insult consist in Dr. Miller's views, in the International Medical Congress, or in both? WOULD he insinuate that Dr. Miller's views insult as expressed as given?? But Dr Miller expressed no views in the last International Medical Congress. No views whatever on dental subjects were expressed in the last International Medical Congress, "as given below." There were no dental members of the last Congress "as given below," or anywhere else. Dr. Miller never was a member of an International Medical Congress, and could not, therefore, have insulted anyone by his views "as expressed as given below." Certainly we never intimated that Dr. Miller had said, as insulted, as the International Medical Congress had expressed as given below—that is in our view—but in your judgment—therefore—whichsoever—

We give it up. The peculiar style of our esteemed critic is beyond imitation. We will no longer dwell upon the lucidity of this clarified Grant White, this reformer of language, this rectifier of journalistic English. King Lear warns us, "O, that my madness lies; let me shun that." We have been endeavoring to comprehend and respectfully to follow this euphonistic purist, but with Macbeth must we cry, "Hence, horrible shadow! Unreal mockery hence." We will return to our own plain and unaffected style, and leave these higher flights to the remodelers of language, the temperance, tobacco, and great moral reformers, whose mission it is to reconstruct the world.

